# Definitive surgery and intraoperative photodynamic therapy for locally advanced non-small cell lung cancer: a case report

**DOI:** 10.1186/s12957-022-02729-5

**Published:** 2022-08-24

**Authors:** Hee Suk Jung, Hyun Jung Kim

**Affiliations:** Department of Thoracic and Cardiovascular Surgery, CHA Bundang Medical Center, CHA University, Seongnam-si, 13496 Korea

**Keywords:** Non-small cell lung carcinoma, Surgical procedures, Photochemotherapy

## Abstract

**Background:**

There are no guidelines for straightforwardly managing advanced lung cancer (T3 or T4). Although surgery has traditionally been regarded as the mainstay treatment and the only curative modality, it has limited relevance for patients with locally advanced non-small cell lung cancer (NSCLC). Photodynamic therapy (PDT) is a clinically approved cancer therapy; it is an established treatment modality with curative intent for early-stage and superficial endobronchial lesions. However, the efficacy of PDT in advanced lung cancer is controversial, and it has primarily been used in palliative care.

**Case presentation:**

This case report describes a 70-year-old male who had right upper lung cancer and an endobronchial lesion that extended into the distal trachea. A biopsy specimen was obtained upon bronchoscopy, and the result confirmed squamous cell carcinoma. We performed a definitive sleeve lobectomy and intraoperative PDT. Gross total resection of the tumor was achieved, but the presence of microscopic residual tumors was inevitable. Complete anatomical resection of the primary tumor by pneumonectomy was not possible due to poor lung function and endobronchial extension to the distal trachea. We decided to apply intraoperative PDT to the lumen and outer wall of the bronchi and distal trachea for local tumor control. The patient is alive with no evidence of disease after 13 months of follow-up.

**Conclusions:**

This is the first report to describe the feasibility and efficacy of intraoperative PDT as part of multimodal therapy for locally advanced NSCLC.

## Background

In photodynamic therapy (PDT), the interaction between light energy and photosensitizers that selectively accumulate in cancer cells is used to eradicate cancer while preserving normal tissues. When energy is absorbed from a light source, the photosensitizers create reactive singlet oxygen and directly cause the necrosis and death of cancer cells. PDT was initially used mainly for skin cancer treatment, but with the development of diagnostic technology and new photosensitizers, the treatment field is expanding to advanced malignant tumors or precursor cancer lesions.

PDT in lung cancer began to be studied in earnest with the development of hematoporphyrin derivatives; in 1980, it was first applied to lung cancer after basic research at the Tokyo University in Japan. In 1982, Hayata et al. reported the outcomes of 16 lung cancer patients treated with PDT [1]. After their announcement that complete remission had been maintained for 4 years after treatment, PDT began to be implemented in early and advanced lung cancers. Clinical experience has expanded considerably since then at centers around the world, which continue to develop treatment indications and new treatment methods based on their own experiences and results [2–4].

Although several recently published trials assessed the role of PDT in treating patients with endoscopically assessable early-stage lung cancer, no studies have investigated the effectiveness of intraoperative PDT in locally advanced non-small cell lung cancer (NSCLC).

### Case presentation

**Fig. 1:**
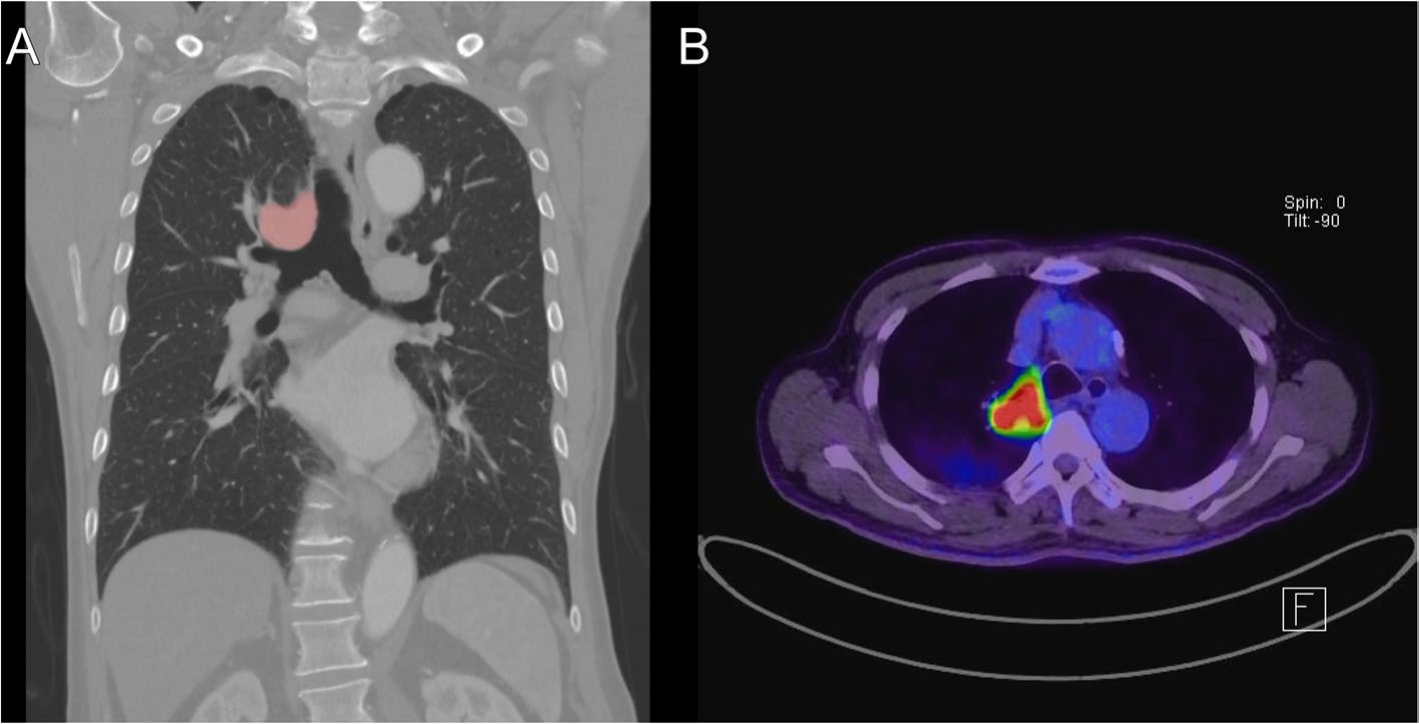
**A** Contrast-enhanced chest CT scan shows an approximately 4 cm mass obstructing the right upper lobe bronchus orifice. **B** PET-CT images reveal a central tumor with increased FDG uptake abutting the right lateral wall of the distal trachea. CT, computed tomography; PET-CT, positron emission tomography-computed tomography

A 70-year-old male patient was admitted to our hospital with dyspnea, cough, and blood-tinged sputum that became aggravated 1 month prior to admission. The patient had been diagnosed with chronic obstructive pulmonary disease 3 years previously and was on medical treatment. He was a 50 pack-year smoker who quit smoking after being diagnosed with emphysema. At the time of admission, his vital signs were stable, but breathing sounds in the right upper lung field were decreased with wheezing on auscultation. Computed tomography (CT) showed a 4-cm lung mass involving the upper lobe, arch of the azygos vein, and mediastinal fat in the paratracheal area. No pleural effusion was present. Bronchoscopy confirmed bronchial stenosis of the upper lobar orifice and an endobronchial lesion that extended into the distal trachea along the lateral wall. A biopsy specimen was obtained upon bronchoscopy, and the result confirmed squamous cell carcinoma. Positron emission tomography (PET) confirmed fluorine-18-fluorodeoxy-D-glucose (FDG) uptake in the right main bronchus, but there was no increased FDG uptake in the mediastinal lymph nodes. Chest CT and PET scan images are shown in Fig. 1. Preoperative respiratory functional tests showed a forced expiratory volume in 1 s of 1.42 L (58% of predicted) and a forced vital capacity of 2.65 L. The clinical stage was T4N0M0 (stage 3A).

**Fig. 2:**
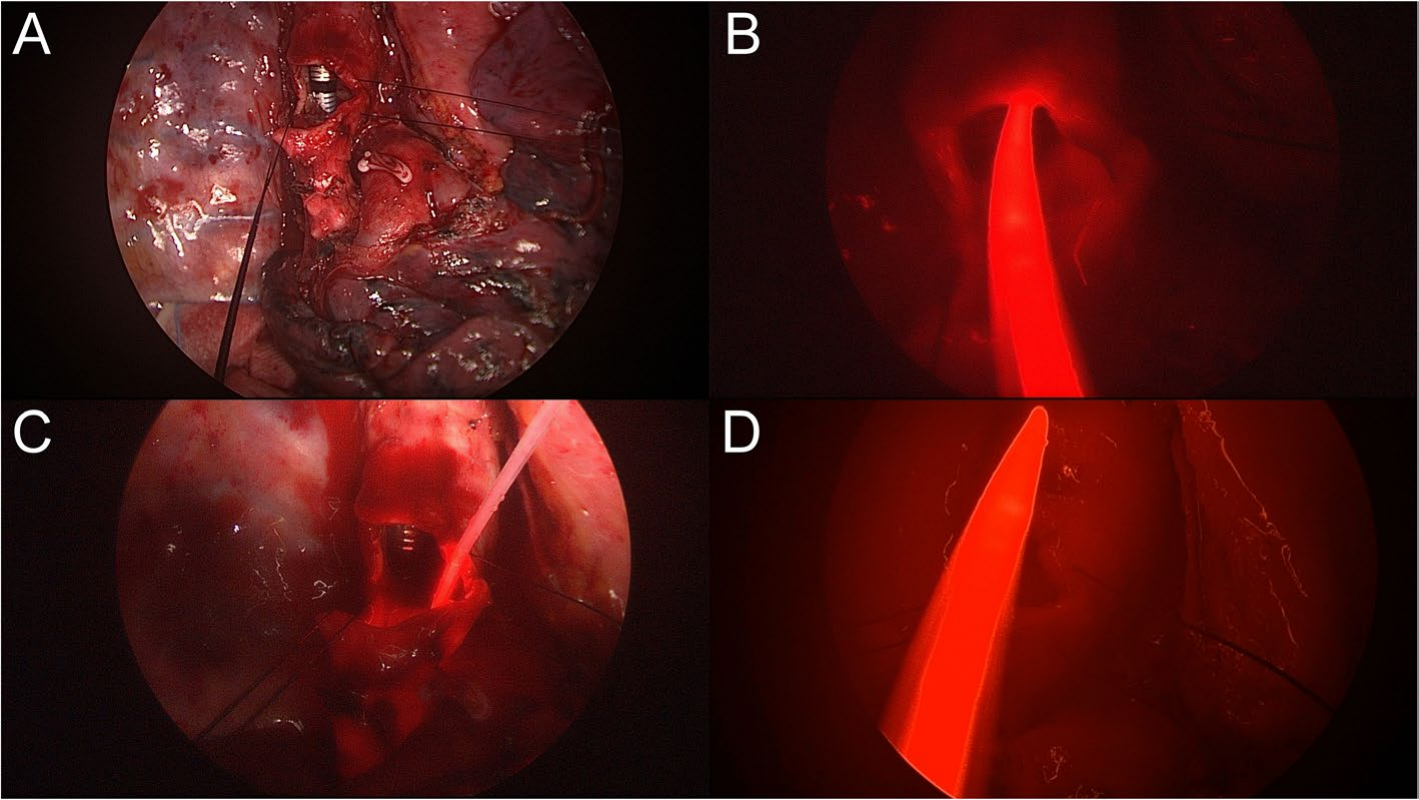
Intraoperative photodynamic therapy. **A** The right main bronchus and bronchus intermedius are divided. **B** and **C** An optical fiber with a 30 mm cylindrical diffuser is placed into the bronchial lumen of the bronchus intermedius and the distal trachea. **D** The laser also irradiates the surgical bed of the tracheal wall to treat microscopic residual tumors

Under general anesthesia with a double lumen endotracheal tube, the patient was placed in the left lateral decubitus position. Through a right posterolateral thoracotomy at the 5th intercostal space, the lung mass was identified in the right upper lobar orifice, encasing the arch of the azygos vein and adhering to the right main bronchus and distal trachea. We decided to conduct a sleeve right upper lobectomy with en bloc resection of the azygos vein. Following circumferential dissection and release from the adjacent anatomical structures, the arch of the azygos vein was divided proximal and distal to the tumor. There was no evidence of tumor invasion into the superior vena cava. The right upper pulmonary artery and vein were dissected and divided using an autostapler device, and complete mediastinal lymph node dissection was performed. Afterward, the right main bronchus and bronchus intermedius were fully mobilized from the pericardium to reduce tension on the anastomosis, and a bronchotomy was performed to assess the precise intraluminal tumor location. The right main bronchus and intermediate bronchus were sharply transected, and the right upper lobe was removed. Although a frozen section of the bronchial margin of the main bronchus was positive for tumor infiltration, further excision of the main bronchial edge was unavailable due to its close proximity to the carina. Because this patient showed distal tracheal invasion on preoperative examination and was expected to have high morbidity or mortality after carinal resection and reconstruction, intraoperative PDT was planned following gross total resection of the tumor. Forty-eight hours before surgery, 2 mg/kg of the photosensitizing agent Photofrin® (Pinnacle Biologics Inc., USA) was mixed with 40 cc of normal saline and injected intravenously. Exposure to sunlight after injection of the photosensitizer was prohibited, but the patient was allowed to live under fluorescent lamp illumination. Before beginning the intraoperative PDT, the chest cavity was irrigated to remove as much residual blood as possible to prevent interference with light delivery. The laser was transmitted via an optic fiber with a 30 mm cylindrical diffuser. Light from a 630 nm laser diode (Diomed, Cambridge, UK) was delivered to the bronchial lumen from the bronchus intermedius to the distal trachea. The surgical bed abutting the tumor was also illuminated before bronchial anastomosis to treat undetected, viable cancer cells. These three sections were each irradiated at 120 J/cm^2^, delivered as 300 mW for 400 s. Figure 2 shows an intraoperative PDT.

**Fig. 3:**
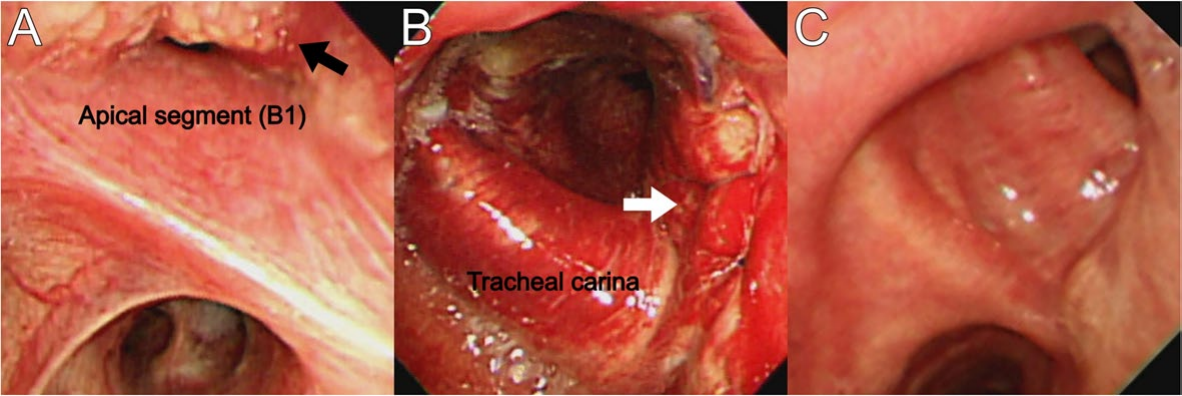
**A** Right main bronchus, a tumor obstructs the right B1 bronchus (black arrow), and superficial invasion extends proximally to the distal trachea. **B** The first bronchoscopy on postoperative day 5 shows an intact bronchial anastomosis (white arrow) with slight bronchial edema and hyperemia. **C** Three months after PDT, the lesion is completely healed by re-epithelization of the normal mucosa. The anastomosis site is slightly narrowed, but the patency is acceptable

A flexible bronchoscopic examination on postoperative day 5 revealed mucosal elimination, and edema could be observed in the area where the PDT was performed. No abnormal findings were seen at the anastomotic site (Fig. 3). The patient was told to avoid exposure to sunlight or bright indoor lights for at least 4 weeks after PDT and was discharged home on postoperative day 8. The day after PDT, he had mild flu-like symptoms, including mild fever and chills, but fully recovered before discharge. No other serious side effects were noted. Postoperative histopathological examination confirmed squamous cell carcinoma of the right lung (T4N0M0, stage 3A). Based on the decision of the multidisciplinary team, the patient received four cycles of adjuvant chemotherapy with a regimen of high-dose methotrexate, cisplatin, and doxorubicin. Bronchoscopy performed 3 months after the surgery showed that the mucosa previously damaged with PDT was almost cured by epithelialization. Only mild stenosis was observed, and no abnormal findings were seen in the biopsy performed at the previously presumed lesions. Thirteen months after the surgery, the patient is alive without evidence of cancer recurrence.

## Discussion and conclusions

Many reports have shown the therapeutic usefulness of PDT in different stages of lung cancer. In palliative settings, PDT effectively reduces airway obstruction and improves respiratory function. Several clinical studies have yielded encouraging results in improving airway obstruction by reducing tumor size. In a retrospective study of 133 patients with airway obstruction caused by advanced inoperable lung cancer, 81% of patients had improved airway patency after PDT [5]. A similar result was demonstrated by Ji et al., who showed that PDT using a second-generation photosensitizer effectively relieved airway obstruction in advanced NSCLC [6]. Although surgical resection remains the standard treatment for early-stage NSCLC, PDT has also been used as a curative treatment in these patients. A recent review of original articles on patients with centrally located early lung cancer treated with PDT found that a complete response was achieved in 30 to 100% of patients, and the overall 5-year survival rate was 61% [7]. PDT might also have an emerging role as a salvage therapy for patients who develop tumor recurrence following surgical resection [8].

In our study, complete anatomical resection of the primary tumor by pneumonectomy was not possible due to poor lung function and endobronchial extension to the distal trachea, so we decided to perform definitive sleeve lobectomy with the intent of removing all visible palpable gross disease and then adding intraoperative PDT to sterilize any microscopic residual disease in the bronchial resection margin and endobronchial lesions. Margin status after surgical resection is an important predictive factor for survival. Postoperative radiation therapy is recommended to improve local tumor control in patients with incompletely resected NSCLC. Neoadjuvant radiotherapy could increase the risk of bronchial stump complications, and the range of the adjuvant radiation field was not expected to be wide enough to include the trachea; therefore, intraoperative PDT was applied as a method for replacing radiotherapy before and after surgery. Postoperative bronchoscopic biopsy of the bronchial stump and tracheal wall revealed no tumor cells, and the patient is still alive without any PDT-related complications. We were able to minimize the loss of pulmonary reserve in the patient by avoiding right pneumonectomy.

Despite the interesting results, our study has limitation of short follow-up period. It is known that recurrence of lung cancer after successful surgery for NSCLC mainly occurs within 24 months [9]. Therefore, it is thought that a longer surveillance period is necessary to clarify the effectiveness of PDT.

PDT is a proven alternative to palliative chemotherapy or radiotherapy in the treatment of advanced lung cancer. It also has a role as a neoadjuvant therapy with or without chemotherapy to reduce the size of tumors for resection. Although a few studies have reported the benefits of intraoperative PDT as part of a multimodal approach to advanced mesothelioma [10], to the best of our knowledge, this is the first case to demonstrate the feasibility and effectiveness of intraoperative PDT in combination with surgery as a curative and lung-sparing modality in the treatment of locally advanced lung cancer. We hope this study will provide relevant evidence-based support for the use of PDT as a method for treating lung cancer.

## Data Availability

The datasets generated and/or analyzed during the current study are publicly available from the corresponding author on reasonable request.

## Abbreviations

PDT: Photodynamic therapy
NSCLC: Non-small cell lung cancer
CT: Computed tomography
PET: Positron emission tomography
FDG: Fluorine-18-fluorodeoxy-D-glucose
